# The Development of an Instrument to Measure the Work Capability of People with Limited Work Capacity (LWC)

**DOI:** 10.1007/s10926-018-9774-x

**Published:** 2018-06-04

**Authors:** Gemma M. C. van Ruitenbeek, Fred R. H. Zijlstra, Ute R. Hülsheger

**Affiliations:** Department of Work and Social Psychology, Faculty of Psychology and Neuroscience, Maastricht University, P. O. Box 616, 6200 MD Maastricht, The Netherlands

**Keywords:** Work capacity assessment, Ability to work, People with limited mental work capacity, Intellectual disabilities, Low literacy

## Abstract

*Purpose* Participation in regular paid jobs positively affects mental and physical health of all people, including people with limited work capacities (LWC), people that are limited in their work capacity as a consequence of their disability, such as chronic mental illness, psychological or developmental disorder. For successful participation, a good fit between on one hand persons’ capacities and on the other hand well-suited individual support and a suitable work environment is necessary in order to meet the demands of work. However, to date there is a striking paucity of validated measures that indicate the capability to work of people with LWC and that outline directions for support that facilitate the fit. Goal of the present study was therefore to develop such an instrument. Specifically, we adjusted measures of mental ability, conscientiousness, self-efficacy, and coping by simplifying the language level of these measures to make the scales accessible for people with low literacy. In order to validate these adjusted self-report and observer measures we conducted two studies, using multi-source, longitudinal data. *Method* Study 1 was a longitudinal multi-source study in which the newly developed instrument was administered twice to people with LWC and their significant other. We statistically tested the psychometric properties with respect to dimensionality and reliability. In Study 2, we collected new multi-source data and conducted a confirmatory factor analysis (CFA). *Results* Studies yielded a congruous factor structure in both samples, internally consistent measures with adequate content validity of scales and subscales, and high test–retest reliability. The CFA confirmed the factorial validity of the scales. *Conclusion* The adjusted self-report and the observer scales of mental ability, conscientiousness, self-efficacy, and coping are reliable measures that are well-suited to assess the work capability of people with LWC. Further research is needed to examine criterion-related validity with respect to the work demands such as work-behaviour and task performance.

## Introduction

In Europe there is growing understanding of the economic and psychological importance of labour participation of people with limited work capacity (LWC). People with LWC concern a very wide and diverse group of people that has, similar to the general population, the right to labour participation stated by the International Covenant on Civil and Political Rights (1966). Unfortunately, fundamental disabilities and restrictions overshadow the talent and work capacity of people with LWC. Their limitations vary from developmental disorders (67%), mental illnesses (19%) and somatic disability (14%), and half of this group deals with a combination of disorders [1]. Although the nature and severity of the limitations differ from person to person, the majority of people with LWC experience difficulties with important cognitive skills that affect the capability to work, such as concentration, memory recall, setting priorities, and problem solving [2]. Furthermore, they often have difficulties with understanding and remembering job related procedures or instructions, interactions with coworkers, lack persistence in order to complete the work, and adapt and act independently (Social Security Administration in [3]). Nevertheless, they are entitled to get the support they need in order to participate in work [4]. Several scholars have highlighted the economic and psychological values of participation in work [5–8]. In addition, more recently research of Schuring et al. [9] showed that people from the target group with a paid job evaluated their mental health, happiness, self-worth and mastery significantly higher than people who stayed unemployed. Moreover, their level of independency increased, while the use of care decreased as a result of labour participation.

Notwithstanding their limitations, the majority of people with LWC are capable to provide a productive contribution dependent on adequate support [10] and a suitable job [11]. We argue that self-report instruments can enable people with LWC to identify their respective strengths and weaknesses and that such an instrument is indispensable for their successful integration into the labour market. However, up to date tailored self-report instruments for selection and support practices of people with LWC are lacking in human resource practices. The existing instruments that have been developed for the general population are not always suitable for people with LWC, since they often deal with low literacy or lack in mental ability to understand the language that is used in most questionnaires. For instance, people with autistic symptoms face problems with metaphorical language. Moreover, people with mental disorders such as attention deficit hyperactivity disorder (ADHD) often face difficulties in concentrating over longer periods of time, which makes it difficult for them to complete extensive instruments. For these reasons we adapted existing instruments in order to develop an instrument that assesses the mental work capability of people with LWC, and that can help to identify their respective strengths and weaknesses. First, such an instrument can inform selection and placement decisions. Second, it sheds light on areas in which people with LWC require training or need specific support on the work floor. Third, it can help people with LWC to reflect on their strengths and weaknesses, which will promote their professional and personal development. As described above, it is crucial that people with LWC are enabled to reflect on their strengths and weaknesses in order to develop a certain level of self-understanding. Timmer et al. [12] claim that self-reflection enables people with LWC to take on more responsibility and initiative, and increases their autonomy. Goal of the present study is therefore to present the development and initial validation of an instrument to assess the mental work capability of people with LWC.

Typically, measures used in Human Resources Management for selection and developmental purposes are self-report measures. Naturally, the validity and usefulness of such measures is dependent on the extent to which individuals are willing and able to reflect on themselves, their feelings, experiences, behaviour, and respond to the respective items. Yet, professionals in the field of integration of people with LWC on the regular labour market, such as job coaches and vocational experts, regularly express concerns that due to their limitations, people with LWC may not be able to critically reflect on themselves and to provide accurate answers in self-report questionnaires. However, we believe that people with LWC will be able to reflect on themselves with the help of tailored measures. For this reason, it is all the more important to tailor the measures to this specific target group and adapt the language. Moreover, to address concerns of professionals in the field, we argue that it is important to use other sources of information in combination with information provided by the target person (the person with LWC). A unique feature of the instrument we developed in the present endeavour is therefore that it consists of a self-report (of the target person) and an observer version that is to be completed by one or more significant others. These are individuals who know the target person well, such as relatives or people who work or have worked with the target person.

In sum, this project aims to develop a customized instrument that measures the mental work capability of people with LWC, in order to enhance the individual support that is given by co-workers or supervisors in the daily work practice on the work floor. As such, the present instrument extends and supplements existing tools with more therapeutic foci (e.g. the Occupational Therapy Practice Framework or Model of Human Occupation). For example, the present instrument can be used as a tool that can facilitate the transition process from clinical support to support in daily practice.

In this study, we combine knowledge from the disciplines of work and organizational psychology and occupational rehabilitation. In doing so, we not only build upon this knowledge but also make important contributions to them. The work and organizational psychology literature has built up a solid knowledge base on how personal characteristics (personality traits, mental abilities) relate to work performance [13–19]. However, this line of research has only considered the general population, while ignoring the specifics of individuals with LWC. In the occupational rehabilitation literature, the specifics of individuals with LWC are well understood, but the role of personal characteristics like personality traits and mental capabilities with employment outcomes has received far less attention [20, 21]. Recently also in this discipline occupational rehabilitation, studies have been conducted on the relation between personality traits and work productivity of people with mental disorders. Nevertheless, we argue that both disciplines can benefit from tailored and validated measures to study more accurately relationships between personality traits and work performance in this specific population.

## Method

Based on the work and organizational psychology literature we first selected specific scales which we expected to be predictive of future work behaviour in our target group. We adapted existing measures of mental ability, conscientiousness, self-efficacy and coping to people with LWC. In the interest of this particular target group, of which the majority deals with fundamental disabilities and restrictions, we chose not only to select predictors from the personality literature (i.e., mental ability, conscientiousness, and self-efficacy), but also a coping measure. Since the nature and severity of the disability can differ from person to person, we deem their coping-style of greater predictive importance than the nature or severity of their disability.

In order to assess the psychometric properties of these customized self-report and observer scales, we follow Hinkin’s [22] steps for scale development. Specifically, we conducted two studies: the first study consisted of two phases. First, we developed the instrument and assessed the comprehensibility of scales for people with LWC. Second, we assessed the psychometric properties such as dimensionality and reliability. In the second study the factorial validity was investigated.

### Study 1: Instrument Development, Dimensionality and Reliability

#### Measures

In this section we describe the theoretical basis for the selection of various scales. The concepts of mental ability, conscientiousness, self-efficacy and coping will be discussed. Furthermore, we will elaborate on the process of assessing the face validity and evaluating the clarity of the language used in the scales for people with a low-literacy level.

General mental ability (GMA) [14] or general cognitive ability [13] refers to individuals’ capability for logical reasoning, solving problems, making decisions, abstract thinking, and the ability to learn [15]. GMA or IQ [23] is generally considered as the most valid predictor of job performance [13–15]. However, the nature of work and its context determine how important mental ability is. For complex tasks, mental ability is often more relevant than for simple tasks. Simple or routinized tasks rely less on problem solving behaviour, and require less abstract thinking and decision making. As a consequence, mental ability has less predictive power for low-complexity than for high-complexity jobs. It goes without saying that all tasks require some level of mental ability, but the required level of mental ability depends on the level of job complexity [15]. An important question in this respect is: which cognitive skills predict work performance of people with limited work capacity? Fadyl et al. [2] argue that cognitive skills that clearly affect the work ability of workers who experience impairment(s) are: attention, concentration, memory, planning and organizing, problem solving, initiation, communication and adapting. The Vocational Cognitive Ratings Scale (VCRS) [24] includes these elements. The VCRS is designed for people with chronic mental illness in order to assess their cognitive strengths and weaknesses in actual work settings, and by that, suggest areas for improvement. We therefore argue that the VCRS can be helpful in our line of research and expect that mental ability measured with an adapted version of the VCRS can be an important predictor of work performance of people with LWC.

Conscientiousness is considered as the second most powerful predictor with respect to work performance in various levels of professions and jobs after mental ability [14, 16, 17]. Barrick and Mount [25] stated that conscientiousness reflects all traits that are important to fulfil all kinds of task in all kinds of professions. People with high levels of conscientiousness are seen as trustworthy, careful and cautious, have high orientation to accomplish tasks [26], are reliable and goal-oriented [16], responsible, and hardworking [25]. Since we seek to identify powerful predictors of success at work in various low level work settings and jobs, conscientiousness seems to be precisely that personal characteristic that is essential for the success in work of people with LWC. In this study the Dutch HEXACO personality inventory [27] was tailored to people with LWC.

Various researchers have indicated that self-efficacy is an important predictor of work behaviour and other important work related outcomes, such as job performance [18, 19]. However, this has only been studied in the general population. Self-efficacy reflects an individuals’ tendency to rely on one’s ability to meet job demands in different work contexts [19, 28, 29]. Self-efficacy can be seen as trust in one’s effectiveness. The self-efficacy theory of Bandura [30, 31] assumes that efficacy determines the type of action people take, the level of effort they put in and their persistency [28]. We think that in particular the level of effort that people are willing to invest and their persistency are important predictors of work success of people with LWC. In this study we adapted the GSES-12 scale of Bosscher [28] to people with LWC.

Coping refers to the cognitive and behavioural effort that people display in order to control, bear or reduce the effects of internal or external stressors [32, 33]. It is an action that is triggered as a result of the (re)appraisal of stressors [32]. Coping can be seen as a dynamic and continuous process of self-regulation. It encompasses actions that are undertaken on a daily basis to master or reduce the impact of any kind of threat (i.e. disease, disorder or limitation) [34]. Finding and in particular keeping a job are to a considerable extent dependent on the effectiveness of the self-regulation or coping strategies of people with serious mental illness [35–37]. Other authors have also indicated how important self-regulation or self-management are as predictors of the development in job-performance [38]. Although these studies have been conducted in the general population we think that coping is an even more important predictor of success for people with LWC, since many of them are dealing with serious restrictions and disabilities they have to overcome. For that reason, the shortened coping inventory for stressful situations (CISS-21) [39] was adopted and adjusted for the use with people with LWC.

After the selection of the concepts described above, we took into account the general guidelines for item development [22]. Statements were formulated as simple and as short as possible, and translated to a language level that is intelligible for people with LWC. Since a large part of people with LWC struggles with low-literacy and/or lacks the mental ability to understand complex language that contains figurative language or double negatives, existing rating scales for mental ability [24], conscientiousness [27], self-efficacy [28], and coping [39] were adjusted to meet a low-literacy level. Items were formulated at language level B1 (simple Dutch), figurative and non-literal language was avoided and items address only one single issue to assure appropriate interpretation of items by the respondents.

A pre-test was conducted to assess the adequacy of the scales and to test whether people from the target group were able to read and interpret the items correctly. First, the relevance for practice and the suitability of the language level of the questionnaire for the target group was discussed in two focus groups consisting of professionals in the field, such as job coaches and vocational experts. Second, 16 people from the target group completed the questionnaire individually under supervision of the first author. In order to check their ability to read the items, we asked them to read questions out loud. To test the correct interpretation, we asked them to explain the meaning of randomly chosen questions. Moreover, people from the target group were also asked how they experienced the completion of the questionnaire, and what their opinion was about the readability and appropriateness of the questionnaire. Feedback from these processes has been incorporated in the questionnaires. Examples of changes made based on the feedback from professionals in the field concerned; explication of what is meant by “organizing work efficiently”. We split this item up into several items referring to concrete actions, such as: “I prepare things, before I start my work”, “I complete tasks in a logical order.”, “I check whether I have done my work correctly.”, and “I correct my mistakes.”

Minor changes have been made in the language of the questionnaire. For example: “I’d rather do something *spontaneously*, instead of working according to a set plan.” has been changed in: “I’d rather do something *as it comes to my mind*, instead of working according to a set plan.”

All original scales discriminate five score options and the majority used five-point Likert-scales. Since a Likert-type scale is most used in behavioural research [22], we chose this type of scaling for all scales. Furthermore, in order to keep answering a questionnaire as easy as possible, all scales were designed in a 5-point Likert scale (1 = never, 2 = sometimes, 3 = regularly, 4 = almost always, 5 = always).

#### Participants and Procedure

We administered our survey twice on several schools for youngsters of our target group: schools for special education (*N* at T_1_ was 35 and *N* at T_2_ was 31 students), schools for practical education and a remedial educational centre (*N* at T_1_ was 75 and *N* at T_2_ was 68 students), and a school for vocational training for low-complexity jobs (*N* at T_1_ was 68 and *N* on T_2_ was 46 students). In total 178 (56.2% male) students participated at T_1_ and 145 (56.6% male) students at T_2_. Participants had a mean age of 17.5 (*SD* = 1.6) at T_1_ and 17.4 (*SD* = 1.5) at T_2_. In total 172 significant others participated at T_1,_ and 136 at T_2_. The significant others who participated varied from parent (*N* at T_1_ was 16, *N* at T_2_ was 15), supervisor (*N* at T_1_ was 11, *N* at T_2_ was 9), mentor (*N* at T_1_ was 127, *N* at T_2_ was 95), to teacher (*N* at T_1_ was 18, *N* at T_2_ was 17).

Participants were informed about the procedure and their rights with respect to the research. If students were above 18 years old and fully accountable, they signed an informed consent themselves. Otherwise, their guardian signed the informed consent. After oral information on the study was given and questions were answered, students completed the questionnaire in a classroom under supervision of the first author. The study was approved by the faculty’s standing ethical committee for psychology of Maastricht University (reference ECP-133- 08_10_2013).

#### Analytic Strategy

In order to statistically test the psychometric properties of the self-report and observer scale and avoiding memory effects, we administered the same questionnaire twice with an interval of 3 months to a group of people with LWC and to a ‘significant other’ of the respondent (such as a parent or mentor). Subsequently, we subjected the data to exploratory factor analysis (EFA) to explore the dimensionality of scales, and we calculated internal consistency of scales and subscales. Furthermore, we determined the test–retest reliability, and we computed the correlation between the scores of the respondent and the significant others. To examine the appropriateness of the adapted scales for people with low mental capacity and low literacy, we did both an exploratory factor analysis (EFA) and assessed the test–retest reliability. We applied EFA to assess whether underlying dimensions of the new scales were consistent with the dimensions in the original scales, and to see whether the dimensionality in both samples corresponded. Moreover, EFA was used to reduce the number of items in order to create a parsimonious set of variables [22]. We separately subjected the items of the different measures (mental ability, conscientiousness, self-efficacy and coping) of the target group and the group of significant others sample to principal components analysis (PCA) a technique for EFA. After inspection of the correlation matrix that demonstrated that components were related, we subjected the conscientiousness and self-efficacy scale to initial PCA with oblique rotation. The mental ability scale and the coping scale were subjected to initial PCA with orthogonal rotation because the component correlation matrix showed that components of both scales were not related. Primarily extraction was based on the factor structure of the original scale, and since the original mental ability scale lacks a clear factor structure the extraction was based on Eigenvalues exceeding Kaiser’s criterion of 1. First, we examined if the Kaiser–Meyer–Olin (KMO) criterion exceeded the acceptable limit of .5 [40] and checked whether the Barletts’ Test of Sphericity reached statistical significance on the different scales in both samples. Subsequently, we examined the correlation matrix on inter-item correlations of variables. A lack of correlation between a variable and other variables justifies deletion of that item [41]. Based on this absence of inter-item correlation we deleted one item from the mental ability scale and one from the conscientiousness scale. After deletion of these items we repeated the PCA procedure. Subsequently, we explored the congruity between the loadings of items on components of the original scales and loadings of items on components of the newly developed scales. When congruity with the original scales was lacking we based the evaluation process about the retention of the number of components on inspection of the scree plots graphs. Additionally, when scree plots showed unclear dimensionality we ran a Monte Carlo Parallel PCA parallel analysis [Watkins, 2000 in [41]. Next, we assessed the test–retest reliability by exploring the relationship between two sets of scores on the scales that were administered twice to the same people at T_1_ and 3 month later (T_2_). We performed preliminary analysis to determine the assumption of normality, linearity and homoscedasticity [41]. We calculated the relationship between the measurements with the Pearsons product-moment correlation coefficient and for the non-parametric correlations Spearman’s rho. We indicated the reliability or the accuracy of scales with Cronbach’s alpha coefficient. Finally, we calculated the self-other correlation in order to explore the accuracy of the observer ratings. Moreover, we explored the self-intimate relationship (e.g. parents, partner, family or friends) correlation, and the self-work-related relationship (e.g. job supervisor, supervisor, personal coach or colleague) correlation. As previous research has revealed differences in the accuracy of observer ratings of personality dependent on the frequency of interacting with targets [42], we explored the self-intimate relationship correlation and the self-work-related relationship correlation. In order to calculate these correlations, we created dummy variables for intimate relationship and for work-related relationship. SPSS version 24 was used for all these calculations.

## Results

Since the original VCRS scale [24] lacks factor structural information we ran an initial PCA on a 23-item scale in which the extraction was based on Eigenvalues exceeding Kaiser’s criterion of 1. Examination of the correlation matrix on inter-item correlations of variables showed no correlation between one item and all other variables in the student sample that justified deletion of this item from the analysis. After deletion of this item and repetition of the procedure, PCA resulted in a five-factor model for the mental ability scale in both samples (i.e. self-report and observer report). These five factors refer to five of the eight cognitive skills that Fadyl et al. [2] recognised as important cognitive skills that can interfere with work functioning such as concentration, memory, planning and organizing, problem solving, and adapting. Moreover, based on congruity between the loadings of items on components in the target group sample and loadings of items on component in the significant others sample, we retained all five components. After inspection of the rotated component matrix we rejected five items, because they had no or relatively low loading on the factor they belong to in terms of content or did not load on a congruent component in both samples. After rejection of those items and repetition of the procedure, the five-factor model with 17 items explained in total 65.35% of the variance in the target group sample, with planning & organisation, learning & memory, adaptability, concentration, and problem solving, contributing ranging from 33.16 to 5.78%. In the significant other sample the model explained in total 72.21%, with variances ranging from 39.67 to 6.16% for the subscales. A more detailed overview of factor loadings is given in Table 1, and a detailed overview of item loadings on components can be obtained from the authors.

**Table 1:** Results of principal component analysis, internal consistency, test–retest reliability and self-other correlation

Scale and subscales	Number of items original scale	Remaining items	Number of components of new scales	Target group sample (*N* = 178)				Test–retest reliability target group	Significant others sample (*N* = 172)				Test–retest reliability significant other	Self-other correlation
				Cumm % of variance	Eigen values	% of variance	α		Cumm % of variance	Eigen values	% of variance	α		
*Mental ability*	23	17	5	65.36			.87	.76**	72.21			.90	.62**	.43**
Planning & organisation	–	7			5.637	33.16	.84	.63**		6.743	39.67	.88	.60**	.39**
Learning & memory	–	4			1.898	11.16	.74	.47**		1.725	10.15	.82	.56**	.25**
Adaptability	–	2			1.372	8.07	.76	.53**		1.233	7.25	.74	.50**	.21**
Concentration	–	2			1.222	7.19	.75	.69**		1.528	8.99	.90	.63**	.28**
Problem solving	–	2			0.982	5.78	.72	.65**		1.048	6.16	.75	.29**	.20**
*Conscientiousness* ^a^	10	8	1	41.26	3.301	41.26	.80	.72**	59.60	4.768	59.60	.90	.78**	.35**^d^
*Self-efficacy* ^b^	12	10	2	50.61			.79	.79**	65.24			.88	.75**	.37**
Persistency	–	6			3.455	34.55	.79	.69**		4.956	49.57	.92	.75**	.32**
Self-confidence	–	4			1.606	16.06	.68	.72**		1.567	15.67	.73	.65**	.26**
*Coping*	21	19 (7)^c^	3	57.19			.85	.67**	80.74			.25	.57**	–
Emotion-oriented	8	8 (2)^c^			4.713	29.46	.88	.75**		1.363	19.47	.79	.57**	–
Task-oriented	7	7 (3)^c^			2.808	17.55	.86	.69**		3.000	42.86	.86	.64**	–
Avoidance	4	2 (2)^c^			1.775	10.19	.64	.68**		1.289	18.42	.69	.53**	–

^a^Originally ten items and four factors: organization (2), diligence (2), perfectionism (3), prudence (3) [27]

^b^Originally 12 items and 3 factors initiative (3), effort (5) and persistence (4) [28]

^c^Number of items significant others scale

^d^The scale is normally distributed in the target group sample and non-normally distributed in the significant other sample, the Spearman’s rho and Pearson correlations are respectively .35 and .36

The internal consistency reliability of the mental ability scale (17 items) was .88 with alphas for the five subscales ranging from .72 to .84 in the self-report scale, and respectively .91 ranging from .74 to .90 in the observer scale.

The test–retest reliability of the complete mental ability scale (17 items) was *r* = .76, with correlation coefficients for the five subscales ranging from .47 to .69 for the self-report scale and respectively *r* = .62 and ranging from .29 to .63 for the observer scale.

The self-other correlation for the mental ability scale was .43, .53 for the intimate relationship and .43 for the work-related relationship, and for the subscales varying from .20 to .39 the self-other correlation, varying from .42 to .62 for the intimate relationship, and varying from .16 to .38 for the work-related relationship.

We executed initial PCA of the conscientiousness scale in which extraction was forced to four components in correspondence with the original scale. Congruity between the loadings of items on components of the original scale and loadings of items on components of the newly developed scale was lacking in both samples. The scree plot showed a clear large drop between the first eigenvalue and the second, followed by a tailing off in both samples, which led to the conclusion that the self-report scales and the observer scale for conscientiousness are unidimensional [43]. A one-factor solution for the conscientiousness scale with 8 items explained in total 37.37% of the variance in the target group sample and respectively 56.55% in the significant others sample.

The internal consistency of the complete conscientiousness scale was α = .80 for the self-report scale and α = .90 for the observer scale. The correlation coefficient of the conscientiousness scale was *r* = .72 for the self-report scale and *r* = .78 observer scale. The self-other correlation for the conscientiousness scale was .35 varying from .46 for the intimate relationship to .34 for the work-related relationship.

We carried out an initial PCA of the self-efficacy scale in which extraction was forced to three components in correspondence with the original scale. Congruity between the loadings of items on components of the original scale and loadings of items on components of the self-efficacy scale was lacking in both samples. The scree plot graphs were unclear, and thus we doubted the dimensionality of the scale. Therefore, we ran additional an Monte Carlo Parallel PCA parallel analysis [Watkins, 2000 in 41], that led us to the conclusion of a two-dimensional model of the self-efficacy scale in both samples. We repeated the PCA procedure in which extraction of two components was forced. Two items did not load on a congruent component in a two factor structured model. For that reason, those items were rejected. A two-factor model with ten items explained in total 50.61% of the variance, with persistency contributing 34.55% and self-confidence contributing 16.06% in the target group sample and respectively 65.24, 49.57 and 15.67% in the significant others sample.

The internal consistency of the self-efficacy scale in total was .79, with alpha for the two subscales ranging from .68 to .79 for the self-report scale and respectively .88 and ranging from .73 to .92 for the observer scale.

The test–retest reliability of the complete self-efficacy scale was *r* = .79 with correlation coefficients’ varying in the subscales between .69 and .79 for the self-report scale and *r* = .75 and from .65 to .75 for the observer scale.

The self-other correlation for the self-efficacy scale was .37, .83 for the intimate relationship and .35 for the work-related relationship, and the self-other correlation for the subscales varied from .26 to .32, varying from .43 to .61 for the intimate relationship, and varying from .24 to .30 for the work-related relationship.

The self-report scale and observer scale differed to a large extent, because we only included observable items in the observer scale. For that reason, the initial self-report scale included 21 items, whereas the observer coping scale included seven items. Nevertheless, we performed initial PCA of the self-report and observer coping scale, extraction was forced to three components in correspondence with the original coping scale. In the observer sample all items loaded in accordance with the three original components of the CISS-21 [39], whereas in the self-report sample only two originally avoidance coping items loaded on the emotion-oriented coping component. We rejected these two items on this conflicting content ground. A repetition of the procedure after rejection of these two items resulted in a three-component solution with 19 items that explained 55.30% of the variance, with emotion-oriented coping, task-oriented coping, and avoidance coping contributing varying from 28.74 to 9.34% for the self-report coping scale. The three-component solution for the observer-report coping scale with seven items explained 80.74% of the variance, with task-oriented coping, emotion-oriented coping and avoidance coping contributing varying form 42.86 to 18.42%.

The internal consistency of the complete coping scale (19 items) was .86, with alpha for the two subscales ranging from .64 to .88 for the self-report scale, and respectively .25, with alpha for the three subscales ranging from .69 to .86 for the observer scale.

The test–retest reliability of the coping scale was *r* = .67 with correlation coefficients varying in the subscales between .68 and .75 for the self-report scale, and respectively *r* = .57 with correlation coefficients’ varying in the subscales between .53 and .64 for the observer scale. Additionally, the inter-item correlation of the four-item avoidance coping subscale was examined because the Cronbach value was smaller than .7. The mean inter-item correlation was .31, which is an optimal inter-item correlation according to Briggs and Cheek [44].

## Discussion

In this first study we executed EFA to assess whether underling dimensions of the new scales were consistent with the dimensions in the original scales, and if the dimensionality of self-reports and observer-reports corresponded. Since there is no factor structure indicated of the original VCRS scale [24] in the literature, the consistency with the original scale could not be assessed. However, results showed similar dimensionality in the adapted mental ability scale in both, the target group sample and the significant others sample.

Although our results indicated that the dimensionality of the adapted conscientiousness scale and the adapted self-efficacy scale do not correspond with the original scales, EFA indicated corresponding dimensionality in the conscientiousness scale and in the self-efficacy scale in both, the target group sample and the significant others sample. Differences in factor structure between the newly developed and the original scales can be explained by the fact that we substantially changed the original scales. We deem the fact that the factor structure show corresponding dimensionality in both samples of greater importance. Furthermore, the factor structure of the self-report coping scale and the observer coping scale corresponded with the original coping scale. Although, the number of items in the self-report scale and the observer scale differ largely, we found similar factor patterns in both samples, and items loaded on corresponding dimensions.

The test–retest correlation of scores on the scales that were completed twice by the same people at T_1_, and at T_2_ indicated low to high correlations for both, the self-report scale and the observer scale. Longer time lags (> 1 month) yield lower test–retest correlations (*r* < .70). Test–retest correlations of > .5 over longer intervals appear to be reasonable for personality traits [45]. Only one self-report subscale and one observer subscale yielded a small test–retest correlation.

Except for two subscales, the Cronbach’s alpha coefficients of the majority of the self-report scales and subscales exceeded .7 indicating good internal consistency [45]. The two exceptions concern two subscales with two and four items respectively, both subscales showed an optimal mean inter-item correlation.

With respect to the observer scales, excluding one coping subscale, all scales and subscales showed internal consistency. The lack of internal consistency of the total coping scale can be explained by the fact that the total observer scale consists only of seven items that reflect three different dimensions of coping. Moreover, two subscales of coping show good internal consistency. And although the Cronbach’s alpha coefficient of the third factor was slightly below the cut-off point of .7, it showed an optimal inter-item correlation.

Finally, we calculated the self-other correlation in order to assess the accuracy of the observer ratings. Even though results show relatively low self-other correlations, this relatively low accuracy level can be clarified by the fact that more than 90% of the observers were work-related observers that accounted for relatively small correlations levels, while only 10% of the intimate-relation observers accounted for medium to large correlations. These results correspond with former research findings. A meta-analysis of Connelly and Ones [42] showed that differences in accuracy in rating personality traits is dependent on interpersonal intimacy, the higher the frequency of interacting with the target, the higher the accuracy.

### Study 2: Testing Factorial Validity

#### Measures

We used the measures for mental ability, conscientiousness, self-efficacy and coping resulting from the above described exploratory factor analysis to test their factorial validity.

#### Participants and Procedure

In order to test the factorial validity new data was collected on several schools (e.g. schools for practical education and a school for low-level vocational training) for youngsters of our target group (20%), and in training centres for work of people with LWC (80%). Questionnaires consisting of the tailored sales for mental ability, conscientiousness, self-efficacy and coping that resulted from study 1, were administered to people from the target group and their significant others. The target group sample consisted of 264 individuals (61.7% male). The mean age of the participants was 26.72 (*SD* = 9.86). The education level of the respondents varied from: 7.6% lack a diploma, 50.8% followed a low level of education, 31.8% finished a secondary vocational education, 2.8% finished higher levels, for 7.2% the level of education is missing. The significant others sample consisted of 221 individuals. Their relation to the target group varied from intimate (59, 5%, such as a parent, partner or family member), personal coach (23.1%), to work related relation (17.2%, such as job coach, work supervisor or internship supervisor).

#### Analytic Strategy

In order to assess the quality of the factor structure, we applied confirmatory factor analyses (CFA) on the new data using Mplus Version 7.2. The CFA procedure consisted of an interactive process in which we evaluated the measurement models resulting from study 1 by examination of fit indices, such as the Chi square test, the Comparative Fit Index (CFI), the Tucker–Lewis Fit Index (TLI, also known as the Non-Normed Fit Index NNFI), the root mean square of approximation (RMSEA) and the standardized square residual (SRMR). If necessary we revised the models based on modification indices that derived from analyses, and afterwards, we re-evaluated the effects of the modifications.

## Results

We performed CFA to cross-validate the five-factor structure of the mental ability scale. Examination of fit indices indicated a reasonable fit for the self-report scale (*N* = 260) (see Table 2). However, inspection of the modification indices indicated that a better fit could be obtained by inclusion of a residual covariance to the model. We accepted this residual covariance because both items are largely similar (‘I know which task is most important.’ and ‘The most important task I do first.’). The model fit indices improved influential after this adaptation: Chi square test χ^2^ (108, *N* = 260) = 192.48, *p* = .000, CFI = .949, TLI = .936, RMSEA (90% CI) = .055 (.042–.067) and SRMR = .046. The fit indices primarily showed also a reasonable fit for five-factor structure the observer scale of mental ability. After inspection of the modification indices, we included the same residual covariance included as we allowed in the self-report scale. The model fit indices improved slightly after these adaptations: Chi square test χ^2^ (108, *N* = 221) = 227.81, *p* = .000, CFI = .942, TLI = .927, RMSEA (90% CI) = .073 (.059–.086) and SRMR = .046.

**Table 2:** Fit indices confirmatory factor analysis

Scale	Sample	Model	Chi square test χ^2^	df	CFI	TLI	RMSEA (90% CI)	SRMR
Mental ability	Target group (N = 260)	Model derived from EFA	241.86^a^	109	.920	.901	.068 (.057–.080)	.049
		Modified model	180.83^a,g^	107	.956	.944	.052 (.038–.064)	.045
	Significant other (N = 211)	Model derived from EFA	423.05^a^	142	.880	.855	.097 (.086–.108)	.065
		Modified model	227.81^a,g^	108	.942	.927	.073 (.059–.086)	.046
Conscientiousness	Target group (N = 264)	Model derived from EFA	61.17^a^	20	.883	.836	.088 (.064–.114)	.054
		Modified model	28.22^b^	19	.974	.961	.043 (.000–.074)	.041
	Significant other (N = 219)	Model derived from EFA	65.22 ^a^	20	.940	.915	.102 (.075–.130)	.042
		Modified model	36.17^c,g^	19	.977	.966	.064 (.031–.096)	.033
Self-efficacy	Target group (N = 262)	Model derived from EFA	55.45^d,g^	34	.968	.957	.049 (.023–.072)	.048
		Modified model	–	–	–	–	–	–
	Significant other (N = 208)	Model derived from EFA	59.86^e^	34	.972	.963	.060 (.034–.085)	.036
		Modified model	–	–	–	–	–	–
Coping	Target group (N = 264)	Model derived from EFA	467.17^a^	149	.826	.800	.090 (.081–.099)	.107
		Modified model	164.29^a,g^	100	.952	.942	.049 (.035–.063)	.068
	Significant other (N = 221)	Model derived from EFA	15.13^f^	11	.993	.986	.041 (.000–.087)	.034
		Modified model	–	–	–	–	–	–

^a^*p* = .000; ^b^*p* = .079; ^c^*p* = .010; ^d^*p* = .012; ^e^*p* = .004; ^f^*p* = .567

^g^χ^2^/df ≤ 3.0

Subsequently, we conducted CFA to test one-factor structure of the conscientiousness scale. The fit indices primarily indicated a poor fit in the self-report scale (*N* = 264). After inspection of the modification indices, we included one residual covariance to the model. The close relation between the two items could be explained by the fact that these two items were composed of one double-barrelled item in the original conscientiousness scale. The model improved influential after this adaptation: Chi square test χ^2^ (19, *N* = 264) = 27.99, *p* = .084, CFI = .974, TLI = .962, RMSEA (90% CI) = .042 (.000–.074) and SRMR = .041. Primarily CFA showed a poor also for the observer conscientiousness scale. After examination of the modification indices we allowed the same residual covariance as in the self-report scale. The model fit indices improved after the modification: Chi square test χ^2^ (19) = 36.17, *p* = .010, CFI = .977, TLI = .966, RMSEA (90% CI) = .064 (.031–.096) and SRMR = .033.

After performing CFA on the two-factor structure of the self-efficacy scale the fit indices indicated a good fit for both, the self-report scale and the observer scale. The fit indices were respectively for the self-report self-efficacy scale: Chi square test χ^2^ (34, *N* = 262) = 55.45, *p* = .012, CFI = .968, TLI = .957, RMSEA (90% CI) = .049 (.023–.072) and SRMR = .048, and of the observer self-efficacy scale: Chi square test χ^2^ (34, *N* = 208) = 59.86, *p* = .004, CFI = .9782, TLI = .963, RMSEA (90% CI) = .60 (.034–.085) and SRMR = .036.

CFA on the self-report coping scale resulted primarily in a poor fit. However, after inspection of we stepwise removed three problematic items since these items cross-loaded on factors, which indicate that these items did not reflect clearly the underling psychological construct. Moreover, we included one residual covariance to the model. The close relation of these two items could also be clarified by the fact that also these two items consisted of one double-bared item in the original coping scale [39]. The fit indices improved influential: Chi square test χ^2^ (100, *N* = 264) = 164.29, *p* = .000, CFI = .952, TLI = .942, RMSEA (90% CI) = .049 (.035–.063) and SRMR = .068. The goodness of fit indices indicated a good fit of the observer coping scale. Chi square test χ^2^ (11, *N* = 221) = 15.13, p = .567, CFI = .993, TLI = .986 showed mediocre results, RMSEA (90% CI) = .041 (.000–.087) and SRMR = .034.

## Discussion

For the evaluation of the goodness of fit, we examined fit indices such as the Chi square test. The smaller the Chi square, the better the fit [22], small non-significant Chi square values suggest a small misfit, while large significant Chi square values suggest a large misfit. Since the Chi square test is sensitive for the sample size, we verified the fit of the models with a relative high χ^2^ and significant χ^2^ as advocated; we divided the χ^2^ by its degrees of freedom [Kline, 2004 in 46]. All adjusted models demonstrated reasonable fits since the statistic adjusted by its degrees of freedom do not exceed 3.0. Furthermore, additional indices like CFI, TLI, RMSEA, and SRMR were included in our goodness of fit examination. Also these results meet the general guidelines [43–45, 47, 48] and showed well-fitting models for the self-report self-efficacy scale, the observer self-efficacy scale, and the observer coping scale without any adaptation. The two conscientiousness scales showed good fits after minor adaptations. The self-report and observer scales for mental ability, and the self-report coping scale showed reasonable to good fits after relatively few adaptations.

### General Discussion

Labour participation is a necessity for all adults. Therefore, also people with limitations are entitled to participate on the labour market at their own level of capacity. An instrument that can indicate the mental work capability of people with LWC is lacking. Therefore, this study concerned the development of a work capability self-report and observer measure that can outline directions to address support in order to encourage the development of self-reflection of people with LWC and enhance occupational rehabilitation practices.

We conducted two studies. In the first study scales for mental ability, conscientiousness, self-efficacy and coping were selected on theoretical base, and subsequently adapted to the language level of people with LWC. The pre-test yielded face validity and gave confidence that the scales were appropriate for people with LWC. EFA yielded congruent factor structures of the adapted scales in both samples and high test–retest reliability, indicating that people with LWC are equally able to complete the questionnaires as their significant other. Moreover, the scales and subscales that evolved from EFA possess adequate internal consistency and observers accuracy correspond with former research. Based study 1 we concluded the developed scales to be appropriate and reliable measures for people with LWC and their significant other.

Finally, we explored factorial validity in study 2. CFA results indicated that factorial validity was established and demonstrated that measures performed as intended. The modifications in scales after CFA only slightly affected factor loadings, internal consistencies, and the test–retest reliabilities. The final instrument with its psychometric properties is provided can be obtained from the authors.

In sum, these studies yielded high test–retest reliability, adequate internal consistent scales with reasonable to good fitting factor models for both, the self-report scales and the observer scales.

To conclude, we developed reliable well-suited measures that can help people with LWC to reflect on their strengths and weaknesses as a requirement for their personal and professional development. It is an instrument that, in addition to the already existing more therapeutic tools, that is expected to be useful in facilitating the transition from clinical support to support in daily work practice. More specific, this tool can strengthen methodical action of professionals in the field with respect to the individual support of people with LWC. The self-report and the observer questionnaire can be completed online or with paper and pencil. The duration of completion varies within the target group from 15 to 45 min, and for the significant other 15 min on average.

Further research is needed to examine criterion-related validity with respect to the work demands such as work behaviour and task performance.

### Limitations and Future Research

Although this multi-source data- and multi-phase study assured that the adapted scales possess content validity and internal consistency reliability, we were not able to assess convergent and discriminant validity due to the limitations in the level of literacy of the target group. Moreover, this study does not cover the final step for scale validation of Hinkin [22]. Further research is required in order assess criterion-related validity in order to explore if the measures possess predictive validity with respect to work behaviour or work performance.
